# Gut microbiota-based bioassay for the quality evaluation of different species of *Dendrobium* and their therapeutic potential in type 2 diabetes

**DOI:** 10.1186/s13020-025-01107-z

**Published:** 2025-05-06

**Authors:** Junju Zou, Qianbo Song, David Tai Wai Lau, Pang Chui Shaw, Zhong Zuo

**Affiliations:** 1 Guangdong-Hong Kong-Macao New Drug Screening Joint Laboratory, School of Pharmacy, Faculty of Medicine, The Chinese University of Hong Kong, Shatin, N.T., Hong Kong SAR China; 2 Li Dak Sum Yip Yio Chin R & D Center for Chinese Medicine and School of Life Sciences, The Chinese University of Hong Kong, Shatin, Hong Kong SAR China; 3 The Hunan University of Chinese Medicine, Changsha, People’s Republic of China; 4 State Key Laboratory of Research on Bioactivities and Clinical Applications of Medicinal Plants, The Chinese University of Hong Kong, Shatin, Hong Kong SAR People’s Republic of China

**Keywords:** *Dendrobium*, Short chain fatty acids, In vitro fermentation, Gut microbiota, Diabetes

## Abstract

**Background:**

*Dendrobium* (DEN) have been utilized as valuable medicinal resource in China used for millennia for prevention and treatment of various diseases, particularly diabetes via alteration of their disordered gut microbiota.

**Purpose:**

The current study aimed to differentiate the quality of different species of DEN via an in vitro system including gut microbiota fermentation followed by antioxidative and hypoglycemic bioassays.

**Material and methods:**

About 100 g fresh DEN from four species (DEN-5 ~ DEN-8) was extracted with 1L boiling water twice with 1 h each time followed by centrifugation to obtain the supernatants as DEN aqueous extract. About 10 mg/mL of each DEN aqueous extract was fermented with fecal fluid from *db/db* mice for 12, 24 and 48 h, followed by monitoring the changes in total polysaccharide and polyphenol contents, antioxidative, hypoglycemic activities. Additionally, the level of short chain fatty acids and the abundance/diversity of gut microbiota in the DEN fermentation mixture were monitored via GC/MS and 16S rRNA, respectively. Moreover, in vivo hypoglycemic activities of DEN-5 ~ DEN-8 at the dosage of 200 mg/kg once daily for 14 days were also evaluated in *db/db* mice.

**Results:**

DEN-5 ~ 8 varied in the content of polysaccharides (0.23–0.61, w/w) and polyphenol (0.008–0.023, w/w). Based on the free radical scavenging percentage, DEN antioxidative activities ranked as DEN-7≈DEN-8 > DEN-5 ≈DEN-6. Based on the percentage of enzyme inhibition, DEN antihyperglycemic activities ranked as DEN-5≈DEN-6 > DEN-8 > DEN-7. Hypoglycemic activities of DEN-5 ~ 8 in *db/db* mice were in the order of DEN-5≈DEN-6 > DEN-8 > DEN-7. Further correlation analyses of DEN polysaccharides content, DEN polyphenol content, SCFAs formation after in vitro fermentation, antioxidant activities, and hypoglycemic activities found that 1) polysaccharides contents in DEN-5 ~ 8 were positively correlate with total SCFAs generated after the fermentation, α-glucosidase inhibitory capacity, α-amylase inhibitory capacity, and GLP-1 level; 2) polyphenol contents in DEN-5 ~ 8 were positively correlate with ABTS, DPPH, and superoxide anion scavenging capacity.

**Conclusions:**

Our current study for the first time utilized a novel in vitro system to assess the hypoglycemic effects of different species of DEN and indicated that polysaccharides content in DEN was positively correlated with hypoglycemic effect while polyphenol content in DEN was positively correlated with antioxidant activity.

**Graphical Abstract:**

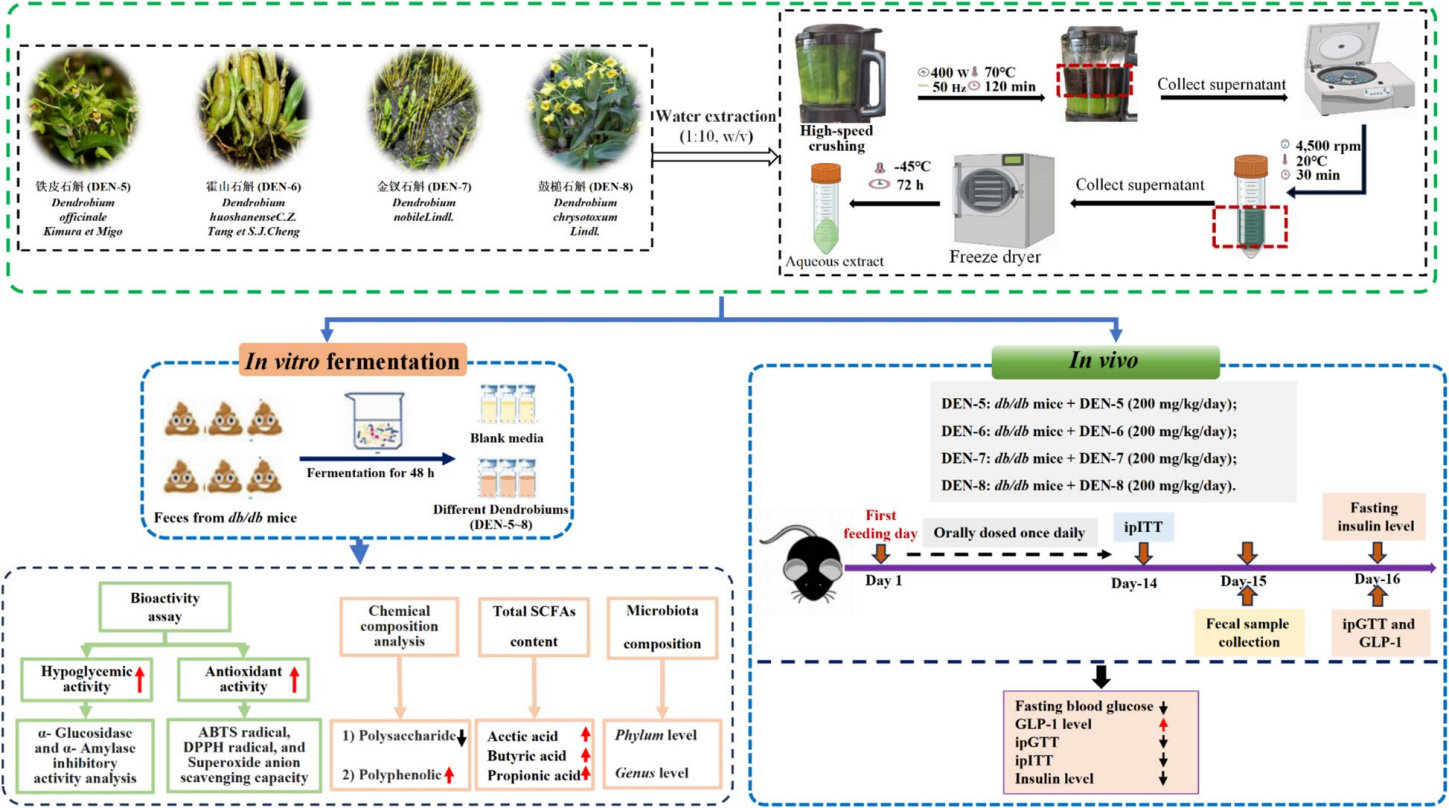

**Supplementary Information:**

The online version contains supplementary material available at 10.1186/s13020-025-01107-z.

## Highlights

- Fermentation of *Dendrobium* in gut microbiota would lead to the increased abundance of healthy bacteria such as *Bacteroides*, *Lactobacillus*, *Ligilactobacillus*, *Bifidobacterium*, and *Akkermansia*, and the abundance of short chain fatty acids.
- Fermentation of *Dendrobium* in gut microbiota could significantly enhance the formation of phenolics and subsequently increased the antioxidative activities.
- *Dendrobium officinale Kimura & Migo and Dendrobium huoshanensis* had more potent hypoglycemic activities and slightly lower antioxidative activity than that of *Dendrobium nobile Lindl.* and *Dendrobium chrysotoxum Lindl.*

**Supplementary Information:**

The online version contains supplementary material available at 10.1186/s13020-025-01107-z.

## Introduction

*Dendrobium* (DEN), an herbaceous species belonging to the Orchidaceae family, has a long history of use in traditional Chinese medicine. According to “Ben Cao Gang Mu”, the most complete and comprehensive medical book of traditional Chinese medicine compiled and written by Li Shi-zhen (1518 ~ 1593), DEN has the potential to enhance “Yin” and augment “Qi”, thereby ameliorating the metabolic dysbiosis of the host [1]. In "Shennong Ben Cao Jing" from Han Dynasty, DEN was documented to improve gastrointestinal function and alleviate symptoms commonly associated with Type 2 Diabetes (T2D), such as excessive urination and thirst [2]. Currently, *Dendrobium officinale Kimura & Migo*, *Dendrobium huoshanensis*, *Dendrobium nobile Lindl.*, and *Dendrobium chrysotoxum Lindl.* are included in the "Chinese Pharmacopoeia" as frequently used traditional Chinese medicines due to their valuable medicinal properties [3, 4]. In addition, DEN juice and DEN tea were developed as functional foods [5, 6].

Over the past decade, extensive studies have revealed that DEN contained multiple bioactive components, including polysaccharides, polyphenol and alkaloids, which demonstrated gastrointestinal modulating [7], anti-tumor [8], immunomodulatory [9], and blood glucose-lowering effects [10]. Recent researches have further demonstrated that DEN was effective in mitigating intestinal injuries and lipid metabolism disorders caused by diets rich in fat, sugar, and alcohol, possibly due to its ability to regulate the gut-liver axis dialogue [11] and modify the intestinal SCFAs-GPR41/43 pathway [12]. Besides, DEN has demonstrated to be able to alleviate high-fat diet-induced nonalcoholic fatty liver via enhancing the growth of probiotics, such as *Lactobacillus*, *Bifidobacterium*, and *Akkermansia* and restoring the gut microbiota balance [13].

Numerous studies have attributed the biological activities of DEN to the polysaccharides and polyphenol present in its stems [14–16]. Since most polysaccharides and polyphenols were poorly absorbed in the small intestine [17], they accumulated in the large intestine and were metabolized by colonic microbiota [18]. Bacteria from gut microbiota have been identified to be able to metabolize polysaccharides and polyphenol to generate phenolic catabolites and short chain fatty acids (SCFA) [19, 20]. Technologies like microbial solid-state fermentation have further been employed to develop DEN-derived products to enhance its biological activities [21–24]*.* Our recent in vivo studies have also demonstrated that the metabolites generated from the gut microbial fermentation of DEN [15] and *Coptidis rhizome* [25] were crucial for their biological activities. Therefore, we hypothesized that an in vitro gut microbiota fermentation system, combined with subsequent bioactivity evaluations, could serve as a tool for assessing quality of DEN.

As the well-established animal model for studying T2D and metabolic disorders, *db/db* mice exhibited gut microbiota alterations that mimic the dysbiosis observed in human diabetes [25]. Although human fecal donors could provide valuable insights, there are practical challenges associated with sourcing and standardizing human intestinal bacteria. To further understand how DEN interacts with gut microbiota under diabetic conditions, the dysbiosis microbial environment in *db/db* mice is believed to be able to provide a more relevant platform. Herein, our current study adopted gut microbiota from *db/db* mice to incubate with four different species of DEN and detect the dynamic alteration of polysaccharides, polyphenol, antioxidative and hypoglycemic activities before and after fermentation. In addition, we also evaluated the effects of four different species of DEN on insulin resistance and glucose tolerance in *db/db* mice. Findings from our studies are expected to provide insights into the assessment of DEN quality from the viewpoint of gut microbial fermentation.

## Materials and methods

### Materials

Four *Dendrobium* species, such as *Dendrobium officinale Kimura & Migo* (DEN-5), *Dendrobium huoshanensis* (DEN-6), *Dendrobium nobile Lindl.* (DEN-7), and *Dendrobium chrysotoxum Lindl.* (DEN-8), were procured from Baoshan City, Yunnan Province; Liuan City, Anhui Province; Chishui City, Guizhou Province; and Baoshan City, Yunnan Province, respectively, and authenticated by Dr. David Tai Wai Lau of the School of Life Sciences, the Chinese University of Hong Kong. Reagents including p-Nitrophenyl-α-d-glucopyranoside (pNPG) (99%), 2,2′-azino-bis (3-ethylbenzothiazoline-6-sulphonic acid) (ABTS) (> 98%), 1,1-diphenyl-2-picrylhydrazyl (DPPH) (> 98%), acetic acid, propanoic acid, and butyric acid were sourced from Sigma-Aldrich (Saint Louis, MO, USA).

### DEN extract preparations

Fresh stems of DEN-5 ~ DEN-8 (100 g) were cut into pieces to obtain a grain size of 0.85 mm and extracted with 1L boiling water twice with 1 h each time followed by centrifugation (4500 rpm, 30 min) to obtain the supernatant as DEN aqueous extract. The obtained supernatant was freeze-dried to yield the DEN extract followed by quantifying the contents of total polysaccharides, monosaccharide composition, and total polyphenol.

### Monitoring of changes in contents and biological activities of DEN during its fermentation with fecal slurry from *db/db *mice

#### In vitro fermentation of DEN-5~DEN-8 with gut microbiota from *db/db* mice

Fresh feces collected from six *db/db* mice (8-week-old) were pooled into a sterile tube and homogenized with autoclaved PBS buffer solution (0.1 M, pH 7.2) at a ratio of 1:10 (w/v) as we described before [15]. The resulting slurry was filtered through double-layer gauze to remove large particles and collect the fecal slurry fluid, ensuring a uniform bacterial suspension. Furthermore, the amount of the bacteria and microbial composition of the fecal slurry was analyzed through 16S rRNA sequencing to confirm the uniformity of the microbial community as we described [15]. The feces collection was approved by the Animal Experimentation Ethics Committee of The Chinese University of Hong Kong (22/008/MIS-5).

For sample treatment, about 120 mg DEN-5 ~ DEN-8 was dissolved in 21.6 mL of sterile anaerobic culture medium consisting of 2.0 g/L peptone, 2.0 g/L yeast extract, 0. 1 g/L NaCl, 0.04 g/L KH_2_PO_4_, 0.04 g/L K_2_HPO_4_, 0.01 g/L MgSO_4_, 0.01 g/L CaCl_2_, 2 g/L NaHCO_3_, 0.5 g/L cysteine-HCl, 0.5 g/L bile salts, and 2.0 mL/L Tween 80. Subsequently, 2.4 mL of the prepared fecal slurry from *db/db* mice was added to these anaerobic mediums. Additionally, 2.4 mL of fecal slurry from C57BL/6 J mice was mixed with 21.6 mL of anaerobic culture medium to serve as a model control for that from *db/db mice*. All cultures were anaerobically incubated at 37 °C for 48 h in an anaerobic incubator (Mitsubishi Gas Chemical Company, Inc. D-119). Samples were collected at 0 h, 12 h, 24 h, and 48 h to assess pH levels and to monitor the dynamic changes in the bioactivity of total polysaccharides and total polyphenol during fermentation involving DEN-5 ~ DEN-8. Moreover, the remaining samples harvested at 48 h were centrifuged at 3500 rpm for 10 min under 4 °C to collect the supernatant for OD_600_ measurement. For those with ΔOD_600_ (OD_600_ sample- OD_600_ blank medium) in the range of 0.6–0.8, their resulting bacterial pellets were retained for subsequent DNA extraction and 16S rRNA gene sequencing as described before [26].

#### Changes in polysaccharide and polyphenolic of DEN during tis fermentation with fecal slurry from *db/db* mice

##### Total sugar content

About 20 mg/mL DEN-5 ~ DEN-8 samples were centrifuged at 4500 rpm for 10 min and the supernatant was subject to sugar content measurement. The total sugar content of DEN-5 ~ DEN-8 was determined by the anthrone-sulfuric acid method using glucose as a standard.

##### Monosaccharide composition analysis

About 20 mg/mL DEN-5 ~ DEN-8 samples were hydrolyzed with 1 mL trifluoroacetic acid (TFA, 2 mol/L) 121 °C for 6 h in a sealed tube. The mixture solution was evaporated to dryness. The residue was re-dissolved in deionized water and reacted with 0.5 mol/L PMP solution for 2 h in basic environment followed by extraction with chloroform. The aqueous layer was filtered through a 0.22 μm filter and 5 μL was injected into the HPLC/UV system for analysis. The chromatographic separation was achieved on an Agilent 1260 HPLC coupled with an Eclipse-XDB-C18 column (5 μm, 250 × 4.6 mm, Agilent) via the mobile phase system of acetonitrile and 20 mM ammonium acetate solution (17:83) at the flow speed of 1 mL/min. The signal was monitored at 250 nm.

##### Total polyphenolic content

The total polyphenolic content of DEN-5 ~ DEN-8 was assessed using a Total Polyphenolics (TP) Detection Kit. Briefly, DEN-5 ~ DEN-8 samples were centrifuged at 4500 rpm for 15 min under 4 °C. The supernatant was then added with 7.5% (w/v) sodium carbonate and Folin-Ciocalteu reagent followed by reaction for 10 min. Gallic acid served as the standard, and results were expressed as milligrams of gallic acid equivalents per gram of dry weight (mg/g).

#### Changes in biological activities of DEN during its fermentation with fecal slurry from *db/db* mice

##### α-glucosidase inhibitory activity

Briefly, different concentrations (0 ~ 20 mg/mL) of DEN-5 ~ DEN-8 and 100 μL of α-glucosidase (1 U/mL) were incubated at 37 ℃ for 20 min. Next, 50 μL pNPG (5 mM) was added and the samples were incubated at 37 ℃ for 10 min. Finally, 1 mL Na_2_CO_3_ (1 mol/L) was added to stop the reaction followed by measuring the absorbance of the mixture at 405 nm by microplate reader (Tecan Spark, Austria).

##### α-amylase inhibitory rate

Different concentrations (0 ~ 20 mg/mL) of DEN-5 ~ DEN-8 samples and α-amylase solution (3.3 mg/mL) were prepared in PBS (0.1 mol/L, pH 6.9) separately. About 500 μL DEN-5 ~ DEN-8 solution was thoroughly mixed with 500 μL of α-amylase solution and incubated at 37 °C for 10 min. Then, 500 μL of a 1% (w/v) soluble starch solution was added to the above mixture for incubation at 37 °C for 10 min followed by termination the reaction by adding 1 mL of DNS reagent and boiling the mixture for 5 min. The obtained was diluted to 10 mL with ultrapure water for measurement of its absorbance at 520 nm by microplate reader.

##### ABTS radical scavenging activity

A solution of ABTS radical was prepared by equal volumes of 2.45 mM potassium persulfate solution and 7 mM ABTS solution. Subsequently, 10 μL of the DEN-5 ~ DEN-8 sample was mixed with 190 μL of the ABTS radical solution and incubated for 6 min. Absorbance of the final mixture at 734 nm was measured by microplate reader.

##### Superoxide anion scavenging activity

About 50 μL of DEN-5 ~ DEN-8 was added to 200 μL of superoxide anion-solution for reaction at 37 ℃ in the dark for 30 min. The absorbance of the final mixture was then measured at 530 nm by microplate reader. Followed by calculating the superoxide anion scavenging activity as follows:

Superoxide anion scavenging activity (%) = (1 − A_1_/A_0_) × 100%, in which A_0_ and A_1_ referred to the absorbances of the negative control (deionized water) and the sample, respectively.

##### DPPH scavenging activity

About 15 μL of the DEN-5 ~ DEN-8 sample was added to 200 μL of DPPH-methanol solution (0.1 mM) followed by incubation at 37 °C in the dark for 30 min. The absorbance of the final mixture was measured at 517 nm by microplate reader to calculate the DPPH radical scavenging activity as follows:

DPPH radical scavenging activity (%) = (1 − A_1_/A_0_) × 100%, in which A_0_ and A_1_ referred to the absorbances of the negative control (deionized water) and the sample, respectively.

#### Changes in microbiota composition after fermentation of DEN with fecal slurry from *db/db* mice

The microbial composition of the fermentation samples collected at 48 h from “In vitro fermentation of DEN-5~DEN-8 with gut microbiota from db/db mice” section were evaluated according to our previous study [15]. Briefly, genomic DNA of the microbe from the sample was extracted using a bacterial DNA isolation kit (Vazyme Biotech Co., Ltd, Nanjing, China). Agarose gel electrophoresis (1%) was used to determine the quality and concentration of the DNA. Additionally, the primers 341F (CCTAYGGGRBGCASCAG) and 806R (GGACTACNNGGGTATCTAAT) were used to amplify the hypervariable V3-V4 region of bacterial 16S rRNA. The Illumina platform was used to sequence the purified amplicons.

#### Changes in pH and short chain fatty acid level during fermentation of DEN with fecal slurry from *db/db* mice

The pH values of the fermented supernatants were determined using a pH meter, while the SCFA level in fecal slurry of was monitored based on our previously established method [15]. Briefly, fermentation samples were centrifuged at 3500 rpm for 10 min at 4 °C. The obtained supernatant (720 μL) was mixed with 1% formic acid solution (80 μL) followed by extraction with ethyl acetate (0.8 mL). The organic phase was obtained followed by being filtered through a 0.22 μm organic-based nylon filter. The filtrate was mixed with trimethylacetate (internal standard, 10 μg/mL dissolved in ethyl acetate) at a volume ratio of 1:1. The obtained solution was subject to analysis of SCFAs levels by 9000 GC–MS system (Agilent, USA) for analysis of SCFAs. In addition, a standard stock solution was prepared by dissolving acetic acid, propionic acid, and butyric acid (each at a concentration of 80 μg/mL) into a blank general anaerobic medium. To establish a standard curve, this stock solution was then serially diluted to create different concentrations followed by the same extraction and analytical procedure mentioned above for the fermentation samples.

### Hypoglycemic effect of DEN extracts in *db/db* mice

#### Animals and treatment

Male mice of 6 weeks aged C57BL/6 J (n = 3) and *db/db* (n = 12) were obtained from Laboratory Animal Services Centre of the Chinese University of Hong Kong. The animal experiment conducted in this study were approved by the Animal Experimentation Ethics Committee of the Chinese University of Hong Kong (Reference No.: 21/167/LDS). The *db/db* mice were randomly divided into five groups (*db/db* group, DEN-5 group, DEN-6 group, DEN-7 group and DEN-8 group, n = 3/per group) and received daily oral gavage treatment for 16 days as indicated below: (1) *db/db* group (*db/db*, distilled water), (2) DEN-5 group (DEN-5, 200 mg/kg/d), (3) DEN-6 group (DEN-6, 200 mg/kg/d), (4) DEN-7 group (DEN-7, 200 mg/kg/d) and (5) DEN-8 group (DEN-8, 200 mg/kg/d). The treatment dosage for DEN was determined to be 200 mg/kg, which was based on our previously identified effective dose of DEN in alleviating T2D symptoms from *db/db* mice [15, 27]. The C57BL/6 J mice served as the control group (C57) and received an equal volume of distilled water during the treatment. Fasting blood was monitored throughout the study in day 0, 3, 7 and 14.

#### Intraperitoneal insulin tolerance test (ipITT)

Mice were subjected to ipITT on day 14. After an 8 h fast, mice were injected intraperitoneally with human insulin (0.75 IU/kg) followed by monitoring the change in blood glucose levels at five time points (0 min, 30 min, 60 min, 90 min, and 120 min) was monitored with a glucometer as we described before [15].

#### Intraperitoneal glucose tolerance test (ipGTT)

IpGTT was performed in all groups on day 16 according our previous study [15]. Briefly, all mice were administered with a 50% glucose solution (0.3 g/kg) intraperitoneally after a 16 h fast followed by collecting blood samples from the tail tip at five time points (0 min, 30 min, 60 min, 90 min, and 120 min) for measurement of glucose levels.

#### Glucagon-like peptide-1 (GLP-1) and insulin level assay

Blood samples were collected on day 17 using EDTA as an anticoagulant followed by centrifugation at 12,000 rpm for 15 min at 4 ℃ to obtain the plasma. Standard solutions of GLP-1 at various concentrations (0, 3.125, 6.25, 12.5, 25, 50 pg/mL) and all plasma samples (diluted threefold) were separately added to a 96-well plate and incubated at 37 °C for 2.5 h. Subsequently, Biotin Conjugate was added to each well and incubated at 37 °C for 1 h. Then, the Streptavidin-HRP solution was added to each well followed by another 45 min incubation at 37 °C. TMB Substrate was then added to each well and incubated for 30 min at 37 °C. Finally, ELISA Stop Solution was added to each well and the absorbance was measured at 450 nm by microplate reader.

### Data analyses

The data were presented as mean ± SEM. The statistical significance of the data was analyzed using Duncan’s test or Student's t-test with a significance threshold of *p* < 0.05. using GraphPad Prism version 9.0. The correlation analyses among changes in gut microbiota, SCFAs, hypoglycemic and antioxidative activities, and polysaccharides and polyphenol from different species of DEN were conducted via Pearson's test.

## Results

### Decreased polysaccharide and increased polyphenolic contents after fermentation of DEN with fecal slurry from *db/db* mice

**Fig. 1:**
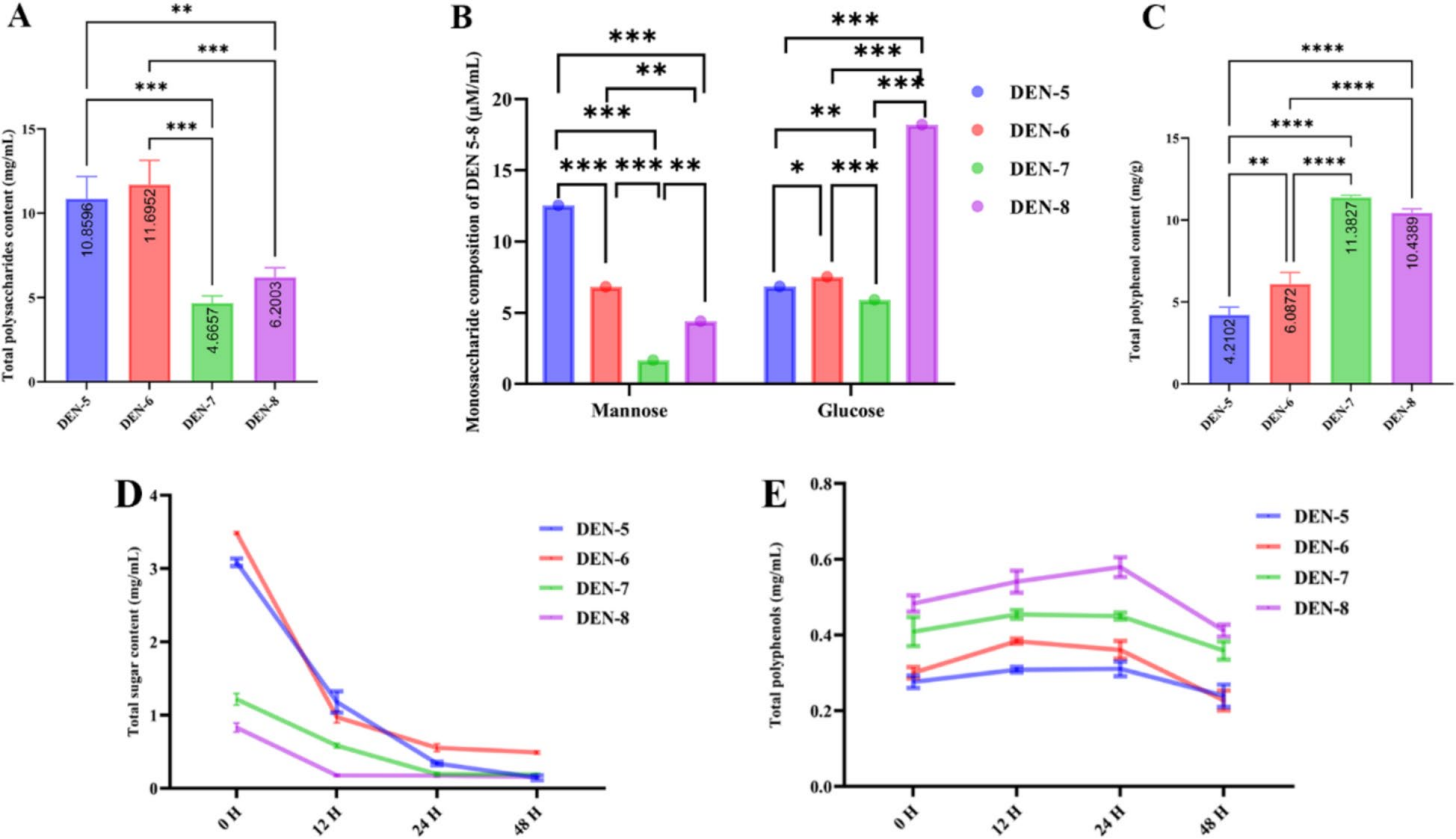
Comparison of total polysaccharides (**A**), major monosaccharides (**B**), and total polyphenols (**C**) in DEN from different species s, alongside the alterations in the levels of total polysaccharides (**D**) and total polyphenols (**E**) subsequent to the fermentation of DEN using fecal slurry from *db/db* mice. *p < 0.05, **p < 0.01, ***p < 0.001, ****p < 0.0001

As shown in Fig. 1A and Table S1, the polysaccharide content of DEN-5 ~ DEN-8 extracts were in the order of DEN-6 (60.73%) > DEN-5 (54.25%) > DEN-8 (31.00%) > DEN-7 (23.33%). The monosaccharide composition of the polysaccharides in DEN-5 ~ DEN-8, as illustrated in Fig. 1B and Table S2, was primarily mannose and glucose, with molar ratios of 12.54:6.84, 6.83:7.52, 1.67:5.91 and 4.4:18.2, respectively. In addition, we also observed that the total phenolic content (TPC) of DEN-5 ~ 8 extracts was in the order of DEN-7 (2.28%) > DEN-8 (2.09%) > DEN-6 (1.22%) > DEN-5 (0.84%) (Fig. 1C and Table S1).

As shown in Fig. 1D, E, it was observed that total polysaccharide content and TPC of DEN-5 ~ DEN-8 exhibited significant alteration after fecal slurry fermentation. The total sugar utilization ratios of DEN-5 ~ DEN-8 were 85.35 ± 1.11%, 86 ± 0.59%, 84.53 ± 0.22%, and 80.85 ± 1.25%, respectively, after 48 h of fermentation with fecal slurry from *db/db* mice (Fig. 1D). Figure 1E showed that the TPC of all DEN-5 ~ DEN-8 significantly increased during the fermentation period of 0–24 h and reached a peak at 24 h, suggesting the promoting effect of in vitro fermentation on the phenolics substance release. After another 24 h of fermentation, TPC significantly decreased.

### Increased hypoglycemic and antioxidative activities after fermentation of DEN with fecal slurry from *db/db* mice

**Fig. 2:**
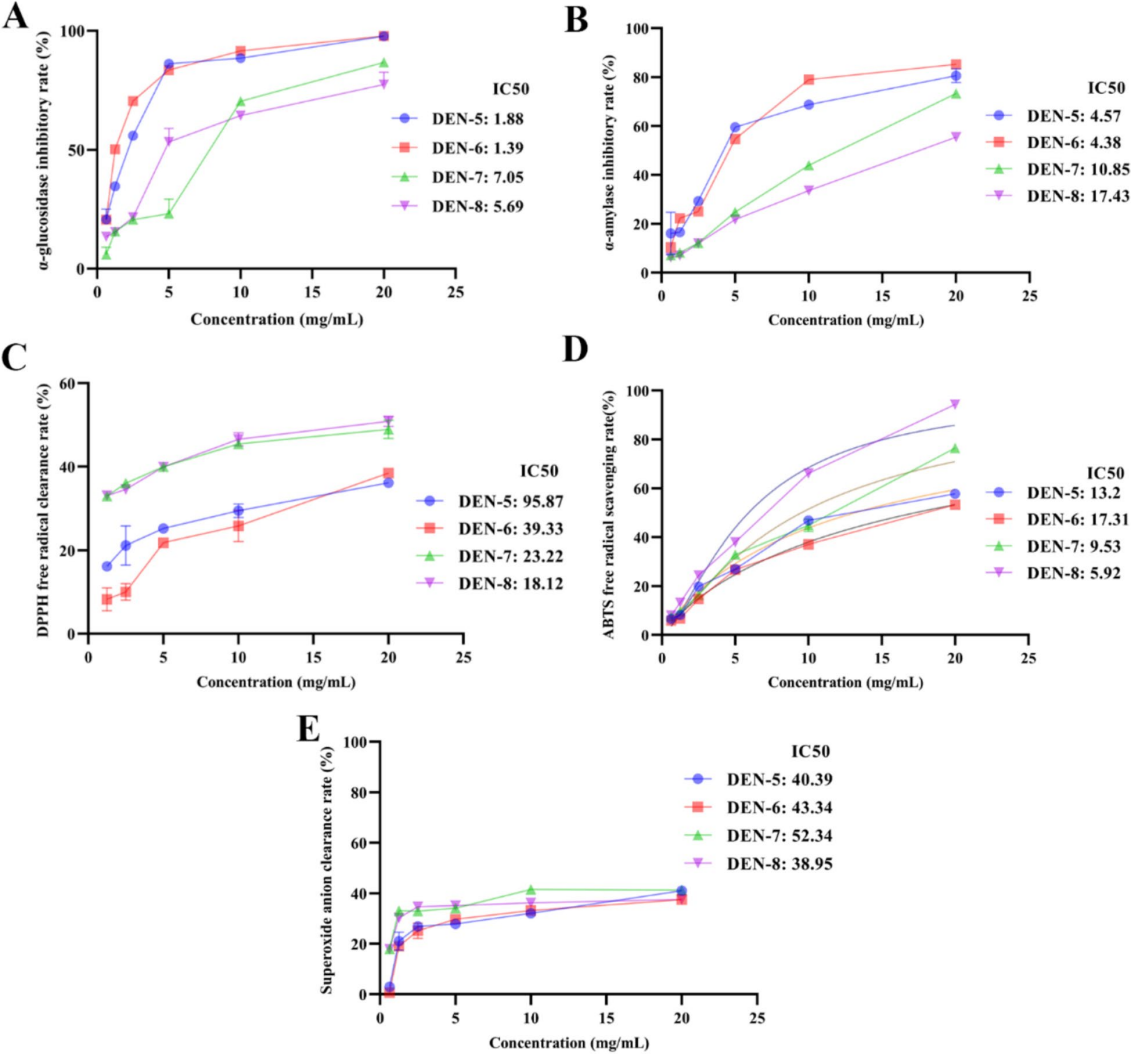
Evaluations of the hypoglycemic [*α*-glucosidase inhibitory activity (**A**), *α*-amylase inhibitory activity (**B**)] and antioxidative activity [antioxidant activities of DPPH radical scavenging (**C**), ABTS radical scavenging (**D**) and superoxide anion scavenging activity (**E**)] in DEN from different species

The inhibition assays of *α*-glucosidase and *α*-amylase by DEN-5 ~ DEN-8 extracts were depicted in Fig. 2 and Table S3. The potency of DEN-5 ~ DEN-8 extracts in inhibiting *α*-glucosidase activity was in the order of DEN-6 > DEN-5 > DEN-8 > DEN-7 (Fig. 2A), while the inhibitory effect on *α*-amylase ranked as DEN-6 > DEN-5 > DEN-7 > DEN-8 (Fig. 2B). Notably, the IC_50_ values of α-glucosidase were significantly lower than those for α-amylase across all tested DEN species. These findings suggested the T2D therapeutic potential for DEN-5 ~ DEN-8.

To evaluate the antioxidative activities of different species of DEN, a series of in vitro assays were conducted (Fig. 2C–E). As shown in Fig. 2C, DEN-5 ~ DEN-8 extracts exhibited DPPH radical scavenging capacity with the IC_50_ values of 95.87 ± 1.98, 39.33 ± 1.59, 23.22 ± 1.37, and 18.12 ± 1.26 mg/mL, respectively. The DPPH radical scavenging capacity of DENs ranked as DEN-8 > DEN-7 > DEN-6 > DEN-5. Consequently, DEN-8 exhibited the most potent ability to scavenge DPPH radicals compared with the other DENs. Figure 2D illustrated the inhibition capacity of DEN-5 ~ DEN-8 extracts on ABTS radicals. Their scavenging capacities of the DENs ranked as DEN-8 > DEN-7 > DEN-5 > DEN-6, with their respective IC_50_ values of 13.20 ± 1.12, 17.31 ± 1.24, 9.53 ± 0.98, and 5.92 ± 0.77 mg/mL. Among the four DEN species, DEN-8 demonstrated the most significant ABTS radical scavenging capacity, with an IC_50_ value of 5.92 ± 0.77 mg/mL. In addition, it was found that DEN-5 ~ DEN-8 extracts exhibited scavenging effects on ABTS radicals at all tested concentrations (1.25 to 20 mg/mL) in a dose-dependent manner. Figure 2E demonstrated that extracts from DEN-5 ~ DEN-8 possessed the ability to neutralize superoxide anions at all evaluated concentrations ranging from 1.25 to 20 mg/mL. Their scavenging capacities were in the order of DEN-8 > DEN-7 > DEN-5 > DEN-6, with IC_50_ values of 40.39 ± 1.61, 43.34 ± 1.64, 52.34 ± 1.72, and 38.95 ± 2.11 mg/mL, respectively.

**Fig. 3:**
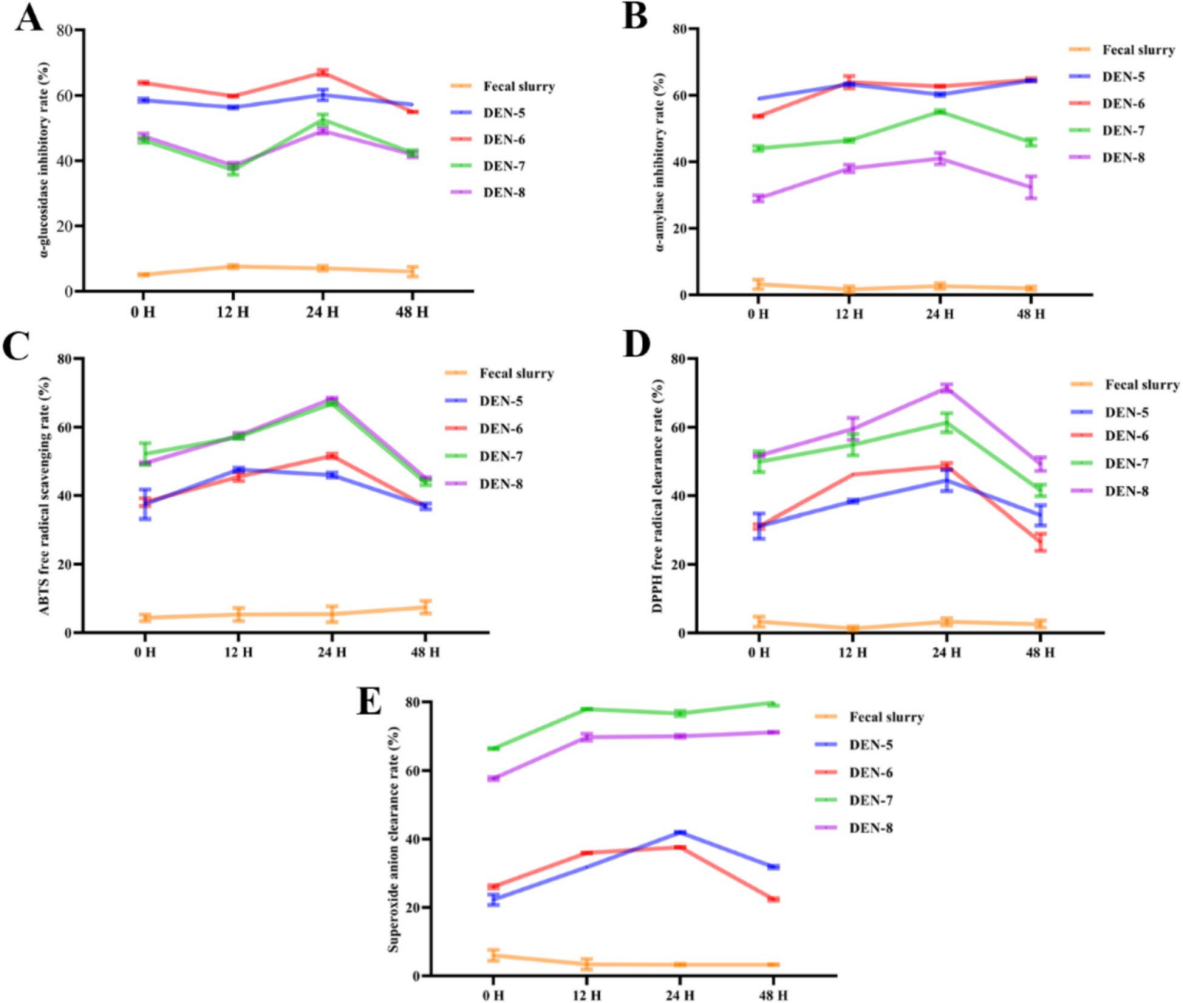
Effect of in vitro fermentations of DEN-5 ~ DEN-8 on the alterations in their contents of hypoglycemic activities [*α*-glucosidase inhibitory activity (**A**), *α*-amylase inhibitory activity (**B**)] and antioxidative activities [ABTS radical scavenging (**C**), DPPH radical scavenging (**D**) and superoxide anion scavenging activity (**E**)]

The inhibitory abilities of DEN-5 ~ DEN-8 on *α*-glucosidase (Fig. 3A) and *α*-amylase (Fig. 3B) reached plateau after 2 h fermentation followed by the decrease in such activities when the fermentation was extended to 48 h. Similarly, the antioxidative capacities of DEN-5 ~ DEN-8 (ABTS clearance capacity (Fig. 3C), DPPH clearance capacity (Fig. 3D), and superoxide anion clearance capacity (Fig. 3E)) also showed the increasing trend in the initial phase (0–24 h) followed by a gradually decline from 24 to 48 h.

### Decreased pH and increased SCFAs formation after fermentation of DEN with fecal slurry from *db/db* mice

**Fig. 4:**
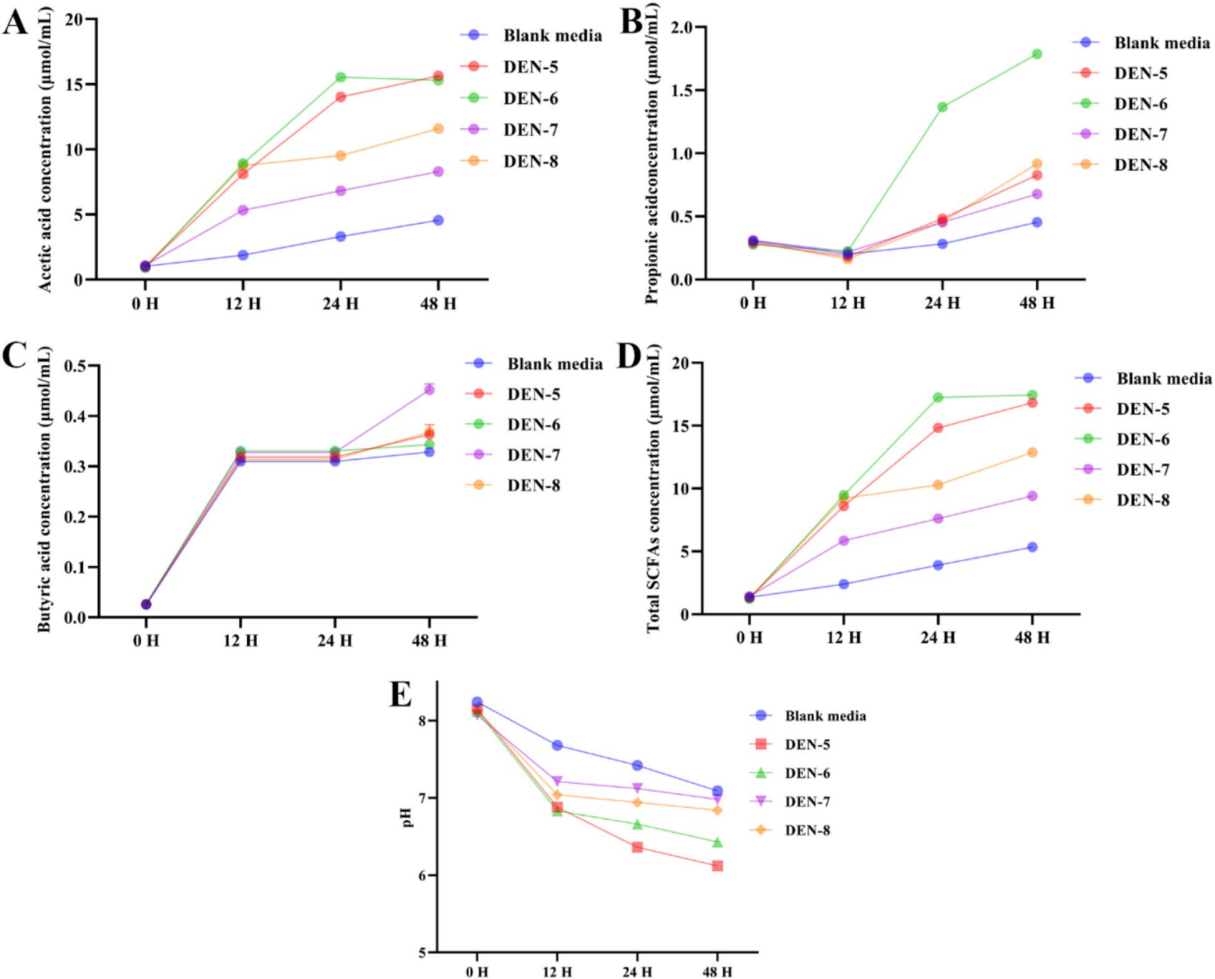
Changes in SCFA concentration [Acetic acid (**A**), propionic acid (**B**), butyric acid (**C**) and total SCFAs (**D**) and pH values (**E**)] during in vitro fermentation of DEN-5 ~ DEN-8

As shown in Fig. 4, the concentrations of SCFAs including acetic acid (Fig. 4A), propionic acid (Fig. 4B), butyric acid (Fig. 4C), and total SCFAs (Fig. 4D) exhibited a gradual increase after fermentation. The total SCFA production at 48 h post fermentation ranked as follows: DEN-5 ≈ DEN-6 > DEN-8 > DEN-7. Additionally, the acetic acid and total SCFA levels in the DEN-5 and DEN-6 groups after fermentation were significantly higher than those in the DEN-7 and DEN-8 groups. Consistently, the decrease of pH was noticed during the fermentation of DEN-5 ~ DEN-8, with DEN-5 and DEN-6 mixtures showing the much lowest pH than that from the rest DENs (Fig. 4E).

### Different alteration in gut microbiota compositions in *db/db *mice by DEN-5~DEN-8 after fermentation of DEN with fecal slurry from *db/db* mice

**Fig. 5:**
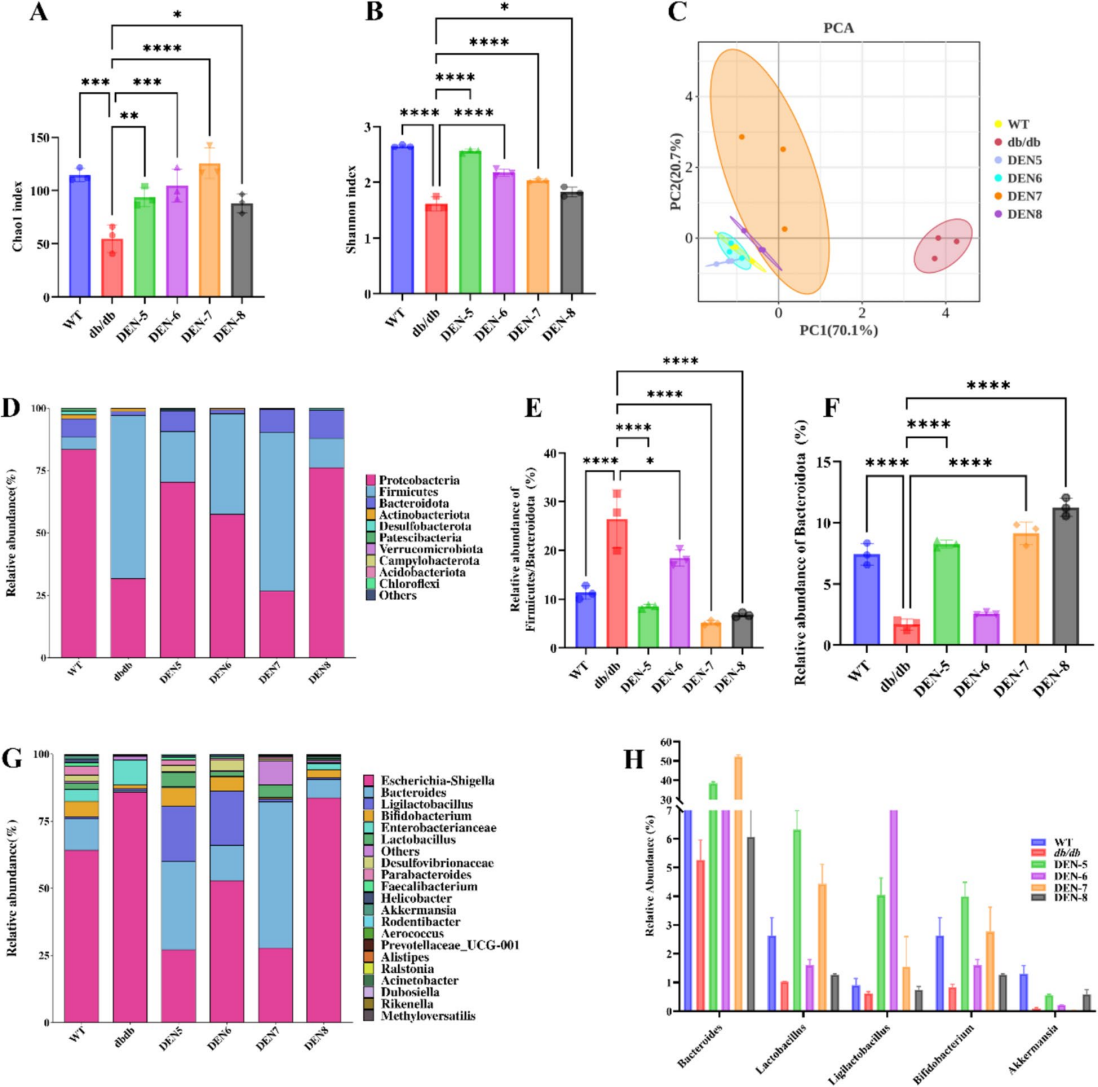
The diversity and composition of microbiota after in vitro fermentations of DEN5-DEN8 with fecal slurry from *db/db* mice as demonstrated by chao1 index (**A**) hannonnon index (**B**), principal component analysis (PCA) (**C**), *phylum* level (**D**), relative abundance of *Firmicutes*/*Bacteroido* (**E**), relative abundance of *Bacteroidota* (**F**), *genus* level (**G**), relative abundance of *Bacteroides*, *Lactobacillus*, *Ligilactobacillus*, *Bifidobacterium* and *Akkermansia* (**H**)

Compared to the *db/db* group, the DEN-5 ~ DEN-8 groups all exhibited increased gut microbiota diversity, as evidenced by the elevated Chao1 (Fig. 5A) and Shannon indices (Fig. 5B), suggesting that the intake of DEN could promote the diversity of gut microbiota. Further, principal component analysis (PCA) indicated that DEN could significantly regulate the gut microbial composition of *db/db* mice (Fig. 5C). To explore the changes in the gut microbiota induced by DEN, we examined the differences in the relative abundance of bacteria at the *phylum* and *genus* levels (Fig. 5D). Compared to that from the *db/db* group, the relative abundance of *Bacteroidota* increased in the DEN-5 ~ DEN-8 groups (Fig. 5E), leading to a decreased *Firmicutes* to *Bacteroidetes* (F/B) ratio (Fig. 5F). The relative abundance of gut microbiota at the *genus* level was shown in Fig. 5G. Compared to the *db/db* group, DEN-5 ~ DEN-8 significantly increased the relative abundance of healthy bacteria such as *Bacteroides*, *Lactobacillus*, *Ligilactobacillus*, *Bifidobacterium*, and *Akkermansia* (*p* < 0.05) (Fig. 5H).

### Verification of different hypoglycemic effect of DEN-5~DEN-8 in *db/db* mice

**Fig. 6:**
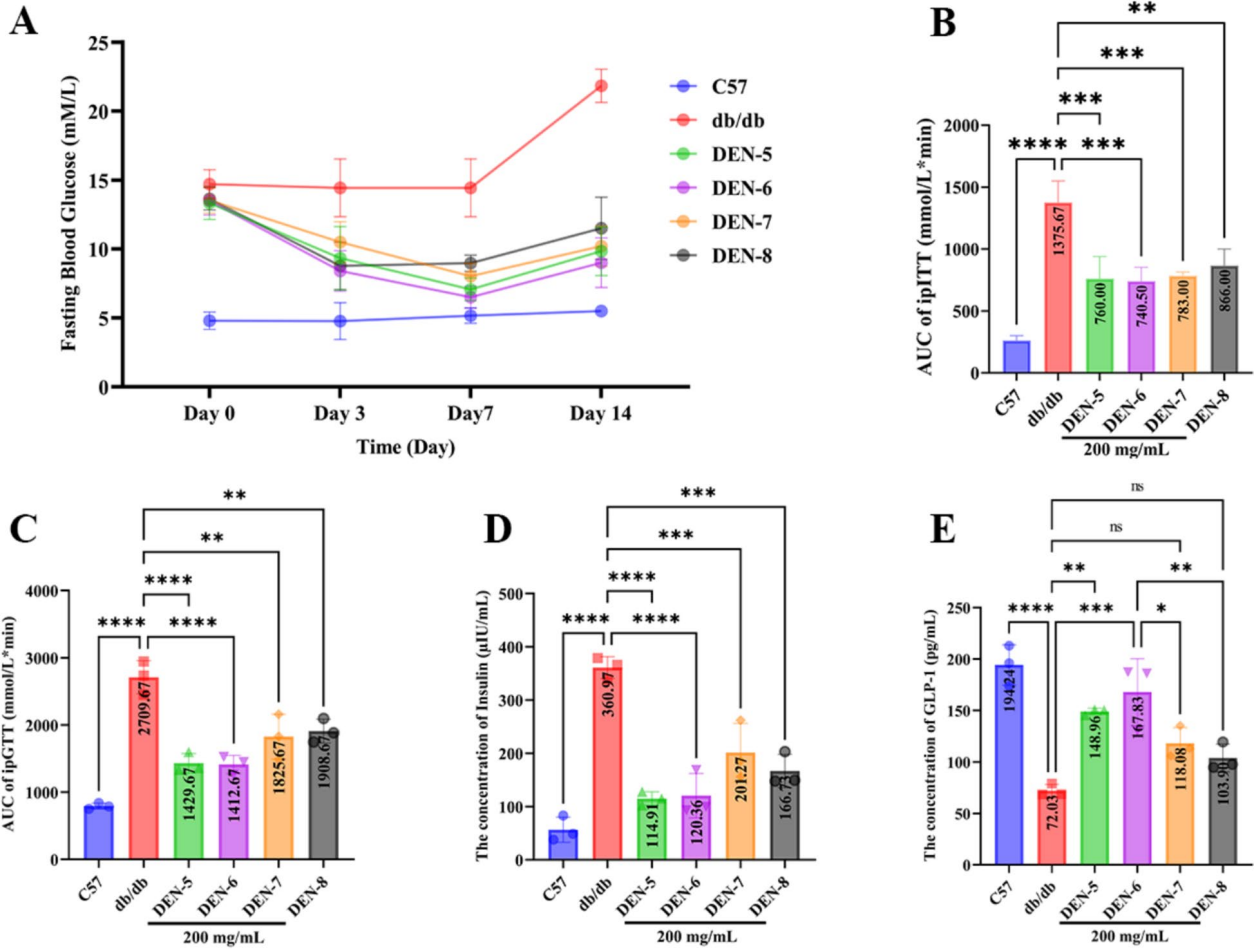
Effect of DEN-5 ~ DEN-8 treatment at 200 mg/kg on blood glucose levels in *db/db* mice indicated by **A** Fasting blood glucose level, **B** AUC of ipITT test, **C** AUC of ipGTT test, **D** Fasting insulin level and **E** GLP-1 level. *p < 0.05, **p < 0.01, ***p < 0.001, ****p < 0.0001

As shown in Fig. 6, compared with the C57 mice, the *db/db* mice exhibited significantly increased fasting blood glucose (Fig. 6A), the area under the curve (AUC) values for ipITT (Fig. 6B) and ipGTT (Fig. 6C), as well as insulin levels (Fig. 6D), whereas the plasma GLP-1 level (Fig. 6E) was significantly decreased. Such phenomenon verified that *db/db* mice exhibited insulin resistance and impaired fasting glucose. After the treatment with DEN-5 ~ DEN-8, the fasting blood glucose, insulin levels, ipITT, and ipGTT in the DEN-5 ~ DEN-8 groups were significantly decreased compared to those of *db/db* group, while plasma GLP-1 levels were significantly increased. Hypoglycemic activities of DEN-5 ~ DEN-8 in *db/db* mice were in the order of DEN-5 ≈ DEN-6 > DEN-8 > DEN-7.

### Correlation analyses among contents of bioactive components in DEN-5~DEN8, gut microbiota alterations, SCFAs formations, hypoglycemic and antioxidative activities

**Fig. 7:**
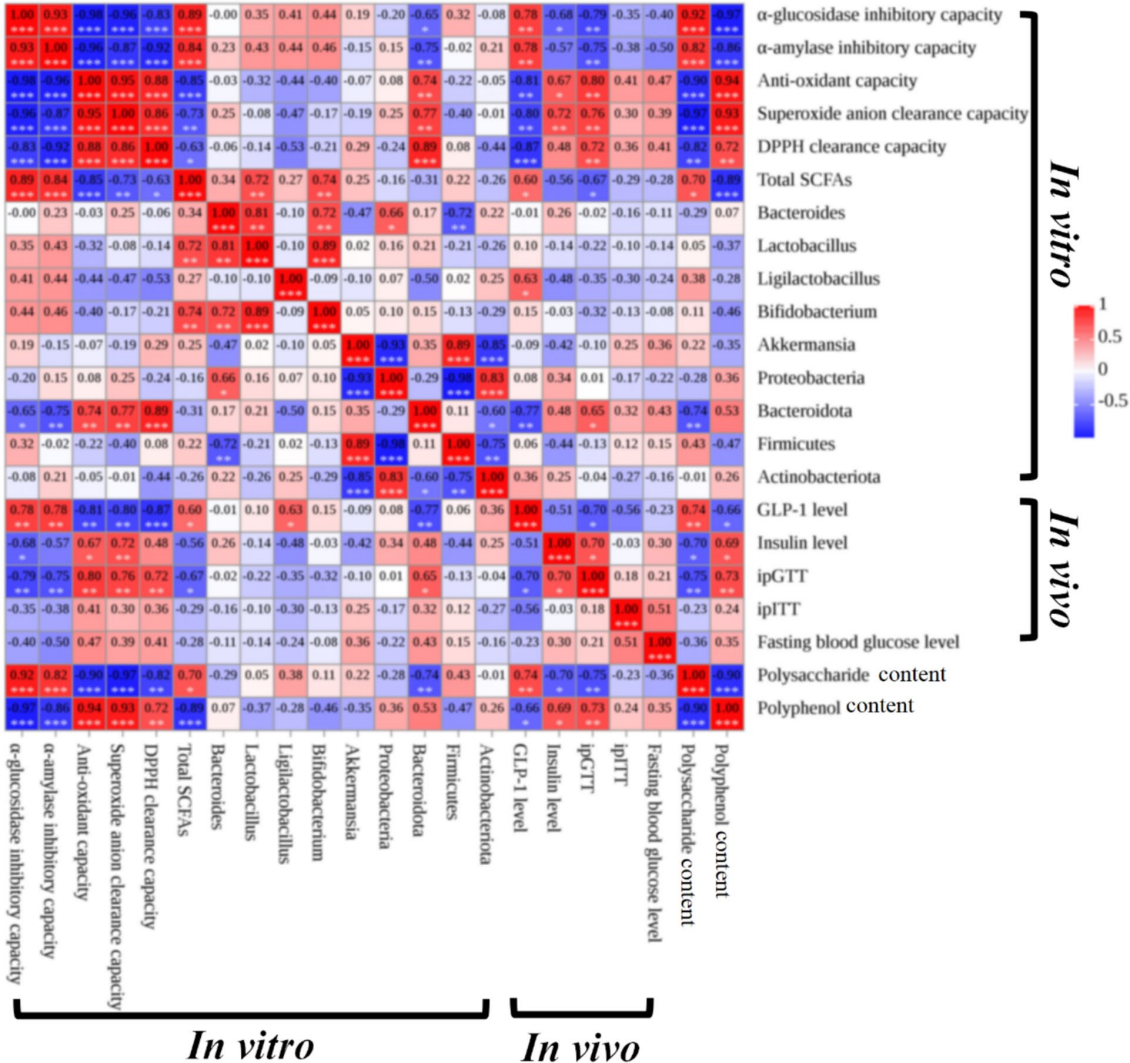
Correlation analyses between contents of polysaccharides and polyphenol, formation of SCFAs, gut microbiota, antioxidative and hypoglycemic activities after in vitro 48 h fermentation, and in vivo hypoglycemic activities of DEN from different species. *p < 0.05, **p < 0.01, ***p < 0.001, ****p < 0.0001

Subsequent correlation analyses among changes in gut microbiota, SCFAs, hypoglycemic and antioxidative activities, and polysaccharides and polyphenol from different species of DEN shown in Fig. 7 revealed that (i) polysaccharide content in DEN-5 ~ DEN-8 was positively correlated with hypoglycemic activities, including *α*-glucosidase inhibitory capacity and *α*-amylase inhibitory capacity after in vitro fermentation, and (ii) polyphenol contents in DEN-5 ~ DEN-8 were positively correlated with their ABTS radical scavenging, DPPH radical scavenging, and superoxide anion scavenging capacities after in vitro fermentation.

## Discussion

**Fig. 8:**
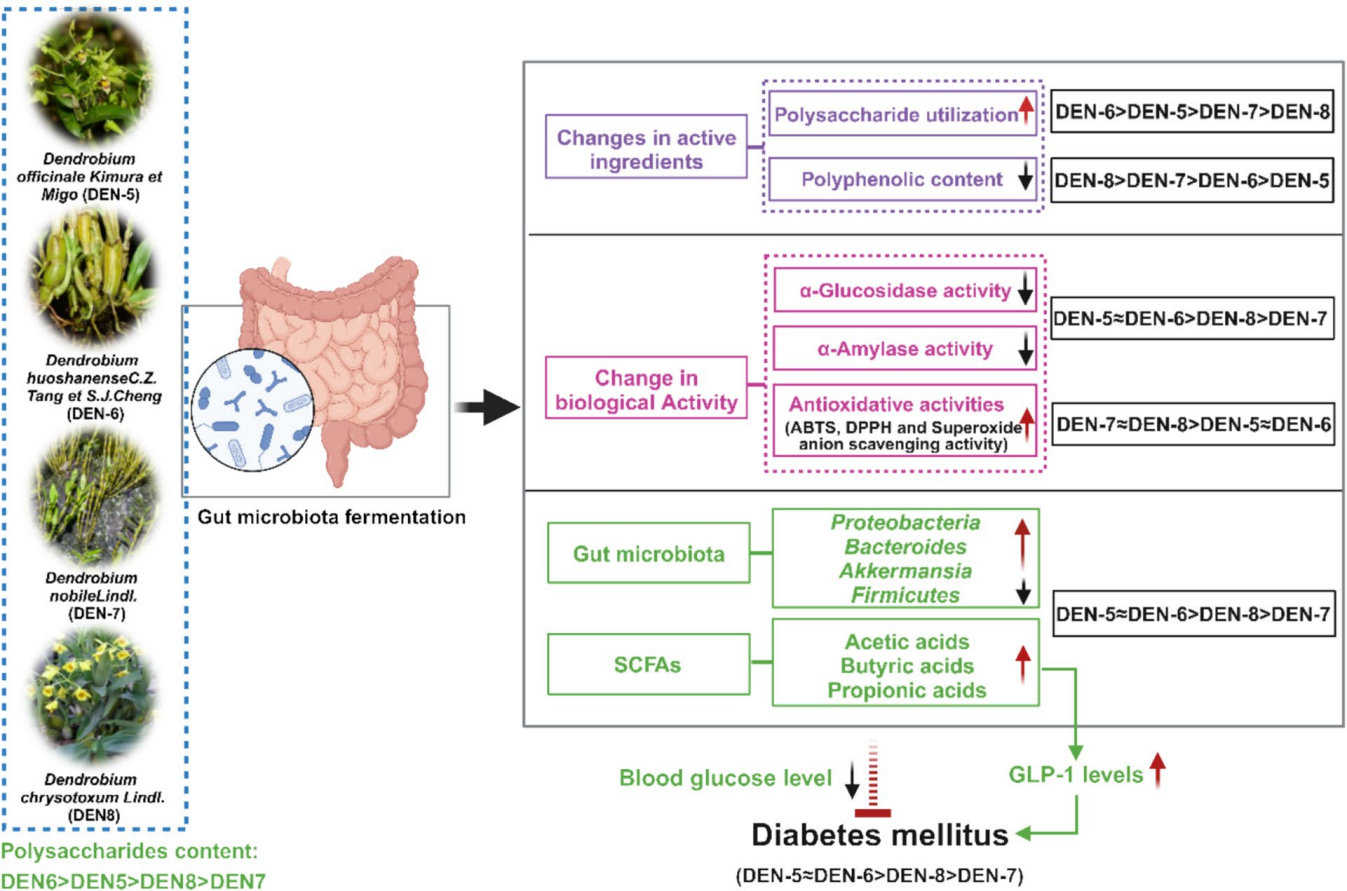
The regulatory role of the gut microbiota fermentation system on the activity of *Dendrobium* from different species

Gut microbiota has been identified as a vital target for many therapeutic drugs including Chinese medicinal herbs [28]. Although DEN have been utilized as valuable medicinal resource in China for millennia to treat diabetes via alteration of their disordered gut microbiota, research on the gut microbiota fermentation of different species DEN remained limited. Our previous study revealed that DEN treatment in *db/db* mice significantly increased the abundance of *Bacteroides* and *Lactobacillus*, with the elevated SCFA production. Such evidence indicated the therapeutic effect of DEN on T2D through regulating gut microbiota and SCFA production [15]. Notably, microbial hydrolysis converted poorly absorbable DEN polysaccharides (e.g., mannoglucans) into oligosaccharides, which underwent further enzymatic biotransformation to generate SCFAs [15]. Such SCFA production process from DEN was also observed in other herbs [29]. Our current study for the first time utilized the in vitro fermentation system to elucidate the application of gut microbiota in quality control of DEN by examining changes in the bioactivity of metabolites during the gastrointestinal metabolism of DEN, the composition of gut microbiota, and the production of SCFAs as highlighted in Fig. 8.

Variations in the species of DEN may lead to changes in the polysaccharide properties including monosaccharide composition, molecular weight, and glycosidic linkage types, all of which could impact their biological activity [30]. Studies had shown that the content and molecular weight of mannose in polysaccharides were closely associated with their immunomodulatory and hypoglycemic effects [31]. This was consistent with our findings that the varying hypoglycemic activities of DEN-5 ~ DEN-8 may be closely related to their polysaccharide content and monosaccharide composition. Previous studies reported that approximately 90% of polysaccharides and polyphenol underwent metabolism and degradation by gut microbiota in the colon [32]. Additionally, it was also observed that the utilized content of polysaccharides in DEN-5 ~ DEN-8 increased with fermentation time, particularly during the initial phase (0–12 h), when utilization was more rapid than during the substantial phase (12–48 h). Furthermore, the polyphenol content of DEN-5 ~ DEN-8 increased following gut microbiota fermentation, which was consistent with previous findings from Chait et al. [33]. Such increase in polyphenol content could be due to the hydrolysis of esterified or conjugated phenolics by gut microbiota [33]. These findings highlight the dynamic alteration of phenolic substances in DEN during its fermentation with gut microbiota.

Maintaining postprandial blood glucose levels was crucial for the early management of diabetes [34]. Inhibition of key enzymes involved in carbohydrate digestion, such as α-amylase and α-glucosidase, was considered as an important therapeutic strategy for diabetes [35]. Our study showed that DEN-5 ~ DEN-8 can inhibit the activities of *α*-glucosidase and *α*-amylase, suggesting potential therapeutic effects on T2D. Plant-derived polyphenols and polysaccharides often exhibit strong antioxidative and free radical scavenging properties, which contribute to maintaining a healthy gastrointestinal tract [36, 37]. Recent studies indicated that polyphenolic compounds extracted from DEN could eliminate reactive oxygen species via hydrogen atom transfer, and free radical adduct formation [38–40]. Our current study also demonstrated that DEN-5 ~ DEN-8 could inhibit the free radical scavenging activity of DPPH, ABTS and superoxide anions. Additionally, the enhancement of antioxidative activity during fermentation may be related to the increased exposure of hydroxyl groups in the system following fermentation by gut microbiota [21].

Gut microbiota plays a significant role in food metabolism and nutrient absorption [41]. Previous studies found that *Akkermansia, Bacteroides,* and *Bifidobacterium* increase acetate production via the acetyl-CoA pathway, promoting GLP-1 and PYY in L cells to lower blood glucose levels in obese and T2D patients [42]. The therapeutic potential of DEN for T2D may be linked to the increased abundance of these bacteria. Additionally, studies reported that consuming orange juice rich in polysaccharides and polyphenol lowered blood glucose and lipid levels by increasing *Lactobacillus* and *Ligilactobacillus* abundance [43]. Our gut microbial analysis revealed that the regulatory effect of DEN on gut microbiota could contribute to its antidiabetic benefits. SCFAs, primary metabolites produced by gut microbiota, helped maintain intestinal microenvironment balance. Propionate could alleviate insulin resistance induced by a high-fat diet and improve insulin sensitivity [44], while sodium butyrate could prevent high-fat diet-induced insulin resistance and improve glucose homeostasis [45]. Acetate and propionate were reported to be able to further enhance insulin sensitivity and glucose tolerance [46]. Therefore, it is suggested that DEN-5 ~ DEN-8 could demonstrate the antidiabetic effects by increasing SCFAs formation, leading to enhanced insulin sensitivity, improved glucose homeostasis, and inhibition of hepatic gluconeogenesis.

Correlation analysis showed positive relationships between polysaccharide content in DEN-5 ~ DEN-8 and hypoglycemic activity after in vitro fermentation, including *α*-glucosidase and *α*-amylase inhibition. Similarly, polyphenol content in DEN-5 ~ DEN-8 correlated positively with ABTS, DPPH, and superoxide anion scavenging activities post-fermentation. Polysaccharides such as *β*-glucan, pectin, arabinoxylan, and galactomannan increase digestate viscosity, delay gastric emptying, and regulate glucose intake [47]. These polysaccharides also exhibited strong *α*-amylase and *α*-glucosidase inhibitory activities [48]. Plant polyphenols, notable for their antioxidant activities, owe this property due to their distinctive polyhydroxy (-OH) structure [49]. These beneficial effects primarily arose from alterations in antioxidant and hypoglycemic activities of polysaccharides and polyphenols during gut microbiota fermentation. It was noted that DEN 7 and DEN 8, which showed strong free radical scavenging capacity, did not exhibit equally strong inhibitory effects on *α*-amylase and *α*-glucosidase compared to that from DEN 5 and DEN 6. Our correlations analyses revealed that the inhibitory activity of the antioxidative effects could be attributed to the abundance of polyphenols in DEN after its gut microbiota fermentation, while the inhibitory effects on *α*-amylase and *α*-glucosidase were attributed to the content of polysaccharides in DEN. The stronger free radical scavenging capacity and weaker *α*-amylase and *α*-glucosidase inhibitory effects of DEN 7 & DEN 8 could be due to their higher polyphenol and lower polysaccharides contents in comparison to that of DEN 5 & DEN 6.

In vivo systems are highly complex and involve multiple physiological processes, such as absorption, distribution, metabolism, and excretion, which could mask the differences observed in vitro by modulating the bioavailability and activity of the metabolites derived from DEN 5–8. Our in vitro study results indicated that DEN-5 and DEN-6 demonstrated higher inhibition rates of *α*-amylase and *α*-glucosidase in the fermentation system compared to that from DEN-7 and DEN-8. Although such trend of efficacy remained similar in their in vivo efficacy evaluations, the differences was not as significant as those observed in vitro due to the potential mask of effect via modulating the bioavailability and activity of the metabolites derived from DEN 5–8 after oral administrations. Although the in vitro gut microbiota fermentation system we developed cannot fully replicate the in vivo metabolic processes and therapeutic effects of DEN, it provides a valuable tool for investigating the gastro-intestinal metabolic transformations of the bioactive macromolecular compounds in DEN, such as polysaccharides and polyphenols.

## Conclusion

Our current study for the first time utilized a novel in vitro system to differentiate the quality of different species of DEN and indicated *Dendrobium officinale Kimura & Migo* and *Dendrobium huoshanensis* had more potent hypoglycemic activities and slightly lower antioxidative activity than that of *Dendrobium nobile Lindl.* and *Dendrobium chrysotoxum Lindl.*. The results inferred that an in vitro system including gut microbiota fermentation followed by antioxidative and hypoglycemic bioassays could serve as a screening tool for assessing the therapeutic effects of different species of DEN on T2D.

## Supplementary Information

Supplementary material 1.

## Data Availability

All data included in this article are available from the corresponding author upon request.

## Abbreviations

DEN: *Dendrobium*
T2D: Type 2 diabetes
SCFA: Short chain fatty acids
DEN-5: *Dendrobium officinale Kimura & Migo*
DEN-6: *Dendrobium huoshanensis*
DEN-7: *Dendrobium nobile Lindl.*
DEN-8: *Dendrobium chrysotoxum Lindl.*
TP: Total polyphenolics
ipGTT: Intraperitoneal glucose tolerance test
ipITT: Intraperitoneal insulin tolerance test
F/B: *Firmicutes* to *Bacteroidetes*
AUC: Area under the curve
GLP-1: Glucagon-like peptide-1
